# Overweight, Obesity and Underweight Is Associated with Adverse Psychosocial and Physical Health Outcomes among 7-Year-Old Children: The ‘Be Active, Eat Right’ Study

**DOI:** 10.1371/journal.pone.0067383

**Published:** 2013-06-25

**Authors:** Amy van Grieken, Carry M. Renders, Anne I. Wijtzes, Remy A. Hirasing, Hein Raat

**Affiliations:** 1 Department of Public Health, Erasmus MC University Medical Center Rotterdam, Rotterdam, The Netherlands; 2 Department of Health Sciences, Faculty of Earth and Life Sciences, VU University Amsterdam, Amsterdam, The Netherlands; 3 EMGO Institute for Health and Care Research, VU University Amsterdam, Amsterdam, The Netherlands; 4 Department of Public and Occupational Health, VU University Medical Center, Amsterdam, The Netherlands; University of Missouri-Kansas City, United States of America

## Abstract

**Background:**

Limited studies have reported on associations between overweight, and physical and psychosocial health outcomes among younger children. This study evaluates associations between overweight, obesity and underweight in 5-year-old children, and parent-reported health outcomes at age 7 years.

**Methods:**

Data were used from the ‘Be active, eat right’ study. Height and weight were measured at 5 and 7 years. Parents reported on child physical and psychosocial health outcomes (e.g. respiratory symptoms, general health, happiness, insecurity and adverse treatment). Regression models, adjusted for potential confounders, were fitted to predict health outcomes at age 7 years.

**Results:**

The baseline study sample consisted of 2,372 children mean age 5.8 (SD 0.4) years; 6.2% overweight, 1.6% obese and 15.0% underweight. Based on parent-report, overweight, obese and underweight children had an odds ratio (OR) of 5.70 (95% CI: 4.10 to 7.92), 35.34 (95% CI: 19.16; 65.17) and 1.39 (95% CI: 1.05 to 1.84), respectively, for being treated adversely compared to normal weight children. Compared to children with a low stable body mass index (BMI), parents of children with a high stable BMI reported their child to have an OR of 3.87 (95% CI: 1.75 to 8.54) for visiting the general practitioner once or more, an OR of 15.94 (95% CI: 10.75 to 23.64) for being treated adversely, and an OR of 16.35 (95% CI: 11.08 to 24.36) for feeling insecure.

**Conclusion:**

This study shows that overweight, obesity and underweight at 5 years of age is associated with more parent-reported adverse treatment of the child. Qualitative research examining underlying mechanisms is recommended. Healthcare providers should be aware of the possible adverse effects of childhood overweight and also relative underweight, and provide parents and children with appropriate counseling.

## Introduction

The prevalence of overweight and obesity is increasing worldwide and has become a public health challenge [1]. The tracking of childhood overweight and associated health consequences into adulthood is of concern [2], [3], [4]. Several serious physical conditions are associated with overweight and, especially obesity, among children including asthma, sleep problems, cardiovascular diseases and type-2 diabetes [4], [5]. Also psychosocial conditions such as lower self-esteem, depressive feelings and body dissatisfaction [6], [7], [8], [9], [10], [11], [12], [13] are associated with overweight or obesity in childhood and adolescence.

A decrease in self-esteem or more depressive symptoms may be caused by weight-related teasing or bullying that may start in childhood and continue into adolescence [10], [11], [13], [14], [15], [16], [17]. Children can experience different types and varying amounts of weight-related teasing [6], [8], [10], [12], [15], [16], [17], [18]. For example, teasing may be peer-related or parent-related and may be daily or sporadic. Moreover, girls and boys report differences in the type and amount of teasing they experience [11].

Most studies have evaluated psychosocial outcomes of overweight and obesity in adolescents and older children (7–12 years) [10], [11], [13], [14], [15], [16], [17] whereas few have evaluated the association between overweight and health outcomes in younger children aged e.g. 5–8 years [18], [19], [20]. Studying the association among younger children, and evaluating changes in weight and potential changes in health outcomes with longitudinal studies, may help to develop appropriate (preventive) interventions for children and parents to deal with both overweight and associated psychosocial/physical consequences.

Similarly, the association between underweight and health outcomes has rarely been assessed, and seldom among younger underweight children [2], [4], [7]. Moreover, ambiguous results are reported on young underweight children and the associations with health outcomes. For example, one study reported decreases in physical health for underweight children compared to normal weight or obese children [4] whereas another reported diminishing associations after controlling for confounding variables [7].

This study aimed to evaluate two questions 1) what are the associations between childhood underweight, overweight and obesity at age 5 years and physical and psychosocial health outcomes at age 7 years, and 2) what is the association between change in body mass index (BMI) measured at age 5 and 7 years and health outcomes at age 7 years.

## Methods

### Study Design

In order to answer both study questions two types of study design were used. For the first study question a prospective design was used, investigating the association between determinants at age 5 years and parent-reported health outcome at age 7 years. For the second study question longitudinal data, obtained at age 5 and age 7, was summarized into one variable defining weight trajectories at age 7 years. Subsequently, a cross-sectional design was used to study the association between weight trajectories and health outcomes both at the age of 7 years. Data to evaluate the study questions originated from the ‘Be active, eat right’ study.

### Study Population

Data was obtained from the ‘Be active, eat right’ study, a cluster randomized controlled trial which aims to assess the effects of an overweight prevention protocol, described in detail elsewhere [21]. The Medical Ethics Committee of Erasmus MC University Medical Center Rotterdam approved the study protocol (reference number MEC-2007-163). A total of 13,638 parents visiting one of the 44 participating youth health care (YHC) centers for their 5-year-old child’s regular preventive health visit between 2007 and 2008 were invited to participate in the study. Of the parents who were present at their child’s health visit, 64.4% provided informed consent (n = 8,784) and 98.9% (n = 8,683) returned a first questionnaire. At 2-year follow-up a questionnaire was distributed among all parents that had given informed consent, for which the response rate was 62.9%.

For this specific study we excluded study participants that were allocated to the intervention condition (n = 4,842) to prevent interference of the intervention with regard to the associations under study. Records with missing data on child height, weight, gender and age at baseline (n = 30) were excluded. In addition, records with missing data for all of the outcomes of interest (n = 1,540) were excluded; these are records from parents that did not return the questionnaire at 2-year follow-up which included the health outcome measures.

Two study populations were created. The first study question, was answered using the population of n = 2,372 children that remained after excluding abovementioned missing data. To evaluate the second study question children with missing data on height, weight, age and gender at age 7 years were additionally excluded; a study population of n = 1,995 remained for the analysis (Figure S1 in the supplementary material presents the data selection process).

### Weight Status of the Child

During the regular preventive health visit, each child’s weight, height and waist circumference was measured by YHC professionals using standardized methods [22]. At 2-year follow-up, research assistants measured child height and weight according to the same methods. BMI was calculated by the researchers as weight in kilograms divided by height in meters squared. Children were categorized into one out of four weight categories according to their age- and gender-specific BMI: underweight, normal weight, overweight (not obesity) and obesity [23], [24].

Children were categorized in weight trajectories to describe changes in weight status. Based on the child’s age- and gender-specific categorization at 5-years and 7-years, children were allocated to one of four trajectories (n = 1,995): low-stable, increasing, high-stable and decreasing. The first trajectory (low-stable) consisted of children maintaining a categorization of normal or underweight BMI, and children moving from the underweight category to the normal weight category or vice versa (n = 1,732). The second trajectory (increasing) consisted of children changing from a normal weight or overweight BMI categorization to the overweight or obesity BMI category (n = 115). The third trajectory (high stable) consisted of children maintaining the categorization of an overweight or obesity BMI (n = 103). The final trajectory (decreasing) consisted of children changing from an obesity or overweight BMI category to an overweight or normal weight BMI category (n = 45). Children changing from an underweight to an overweight or obese BMI category or vice versa were excluded (n = 1).

### Health Outcomes

Data on children’s health outcomes (age 7 years) were obtained by questionnaires completed by parents. Table 1 presents the items assessing the indicators of psychosocial health outcomes. The items measuring psychosocial health outcomes were based on existing studies and developed by the researchers to fit the population under study [25], [26], [27].

**Table 1:** Overview of items providing an indication of psychosocial health.

Item name	Question/assessment	Scoring
Happiness	How often, in the past four weeks, was your child happy?	1 = always to 5 = never
Insecurity	I sometimes feel that my child feels insecure due to his/her weight	1 = totally disagree to 5 = totally agree
Adverse treatment	I sometimes feel that my child is treated adversely due to his/her weight(for example being teased, left behind or ignored)	1 = totally disagree to5 = totally agree
Parental concern	Sometimes, I am concerned about my child’s weight and the consequencesthereof for his/her health	1 = totally disagree to 5 = totally agree

Parents were invited to fill in the number of visits to the general practitioner (GP) due to issues with the child’s weight in the past two years, which was categorized as none versus one or more visits. The presence of common health conditions was assessed by the item: “Does your child have one of the following conditions?”. Parents could choose ‘yes’ or ‘no’ to each of the following conditions: hearing difficulties, seeing difficulties, abdominal pain, headaches or migraine, allergies, and eczema. The presence of asthma symptoms was assessed with the wheezing and dyspnea questions from the International Study of Asthma and Allergies in Childhood (ISAAC) questionnaire [28]: “Has your child suffered from wheezing or a whistling noise in the chest/shortness of breath or breathlessness in the past 12 months?” and “How often in the past 12 months has your child suffered from wheezing or a whistling noise in the chest/shortness of breath or breathlessness?” Answers were dichotomized into ‘symptoms’ versus ‘no symptoms’.

Health-related quality of life of the child was assessed with the General Health scale from the Child Health Questionnaire Parent Form 28 (CHQ-PF28) [29]. The General Health scale was dichotomized [mean: 86.40 (sd: 15.43)] into low scoring (<71.97) or average to high scoring (≥71.97).

### Child and Maternal Characteristics

Information on child gender, age (years) and ethnic background (Dutch, non-Dutch) was obtained at enrolment. Child ethnicity was determined by the parents’ country of birth: if both parents were born in the Netherlands the child was classified as ‘Dutch’, otherwise the child was classified as ‘non-Dutch’ [30].

In our study sample most of the questionnaires were completed by mothers (90.3%). Information on maternal age (years), height (meters), weight (kilograms), country of birth (the Netherlands, other) and educational level was obtained at enrolment. Maternal BMI was calculated as weight in kilograms divided by height in meters squared. Maternal level of education consisted of four levels: low (no education, primary school, or ≤3 years of general secondary school), mid-low (>3 years of general secondary school), mid-high (higher vocational training, undergraduate programs, or bachelor's degree), and high (higher academic education) [31].

### Analyses

Normal weight, overweight, obese and underweight children were compared with regard to baseline child (gender, age, ethnic background, BMI) and maternal characteristics (age, country of birth, education level and BMI) by means of one-way analysis of variance and chi-square tests.

The non-normal distribution of the data required ordinal regression analyses to be performed for the items on happiness, insecurity, adverse treatment and parental concern. Logistic regression models were fitted for all other health outcomes: GP visits, ISAAC items, health conditions, and general health.

For both study questions regression models were fitted. First a model with with BMI in categories at age 5 years as independent variable was fitted; secondly a model with weight trajectories as independent variable was fitted. Underweight, overweight and obese children were compared with normal weight children (reference category) and the high-stable, increasing and decreasing weight trajectories were compared with the low-stable weight trajectory (reference category). All regression models were corrected for potential confounding variables gender, ethnic background of the child and education level of the mother [12].

The ordinal regression model provides the estimated odds ratio (OR) with 95% confidence interval (95%CI) for having a higher score on the outcome variable, if the independent variable would increase with one unit. An OR is estimated for each category of the independent variable compared to the reference category; the model assumes that all other factors stay constant. Tests of parallel lines were performed to check the use of the ordinal regression model. A multinomial regression model was fitted when the parallel lines test was significant; coefficients of the ordinal and multinomial regression models were compared. All ordinal regression models coefficients were in line with the coefficients of the multinomial models.

Effect modification was explored for gender, ethnic background and maternal education level: an interaction term with weight category was added to the above- described models [12]. Interaction terms were evaluated at p<0.10. Wardle et al. [12] recommended presenting results stratified. Due to the number of and inconsistency in the results of the models including the interaction terms, an overview of analyses stratified for child’s gender, child’s ethnicity and maternal education is provided in the supplemental material Table S2.

Demographic characteristics of mothers (age, country of birth, education level and BMI) with missing data on the outcomes (n = 1,540) were compared with characteristics of mothers with no missing data (n = 2,372) by means of descriptive statistics.

Analyses were performed using SPSS version 20.0 for Windows (International Business Machines (IBM) Corp., SPSS statistics, version 20.0, Armonk, New York, USA).

## Results

The mean age of the included children was 5.8 (sd: 0.4) years, 50.0% were boys and 89.9% were Dutch (Table 2). There was a significant difference in the distribution of boys (p<0.001) and non-Dutch children (p<0.001) across the weight categories; more girls were categorized having overweight and more non-Dutch children were categorized as having obesity. Table S1 (available in the supplemental material) presents the baseline distribution of health conditions. Compared to mothers with no missing outcome data, the mothers of children with missing outcome data were younger [mean: 35.6 (sd: 4.6) years vs. 36.7 (sd: 4.2) years; p<0.001], more often non-Dutch (56.1% vs. 37.4%; p<0.05), less often higher educated (32.5% vs. 67.5%, p<0.001) and had a higher BMI [mean: 24.0 (sd: 4.4) vs. 23.7 (sd: 3.7); p<0.01].

**Table 2:** General characteristics of the study population, stratified by children’s weight status (n = 2372).

	Total (n = 2372)	Underweight†(n = 355)	Normal weight†(n = 1830)	Overweight†(n = 148)	Obesity†(n = 39)	p-value*
**Child characteristics**						
Age in years, mean (SD) [missing n = 0]	5.8 (0.4)	5.8 (0.4)	5.7 (0.4)	5.8 (0.4)	5.8 (0.6)	0.396
Gender, % boys [missing n = 0]	50.0	47.9	51.5	37.2	43.6	0.005
Ethnic background, % Dutch[missing n = 34]	89.9	89.2	90.6	87.8	68.4	<0.001
BMI, mean (SD) [missing n = 0]	15.4 (1.5)	13.4 (0.5)	15.4 (0.9)	18.2 (0.7)	21.1 (1.2)	<0.001
**Characteristics of the mother**						
Age in years, mean (SD) [missing n = 251]	36.7 (4.2)	36.6 (4.1)	36.7 (4.2)	36.8 (4.4)	37.6 (5.7)	0.581
Born in the Netherlands, % yes[missing n = 2]	92.9	93.0	93.6	88.5	74.4	<0.001
Educational level [missing n = 6]						
% low	3.0	2.0	3.1	3.4	5.1	0.050
% mid- low	16.7	17.2	16.0	20.9	28.2	
% mid- high	45.6	45.4	45.2	52.0	46.2	
% high	34.7	35.5	35.8	23.6	20.5	
BMI mean (SD) [missing n = 64]	23.7 (3.7)	23.0 (3.9)	23.6 (3.5)	24.8 (4.5)	27.9 (5.6)	<0.001

† Categories based on international age- and gender-specific BMI cut-off values.

* p-value from Chi-square tests for categorical variables and ANOVA for continuous variables, comparing general characteristics across weight categories.

### Associations between Underweight, Overweight or Obesity at Age 5 Years and Health Outcomes at Age 7 Years

Table 3 presents the results of the regressions analysis predicting health outcomes at age 7 years with BMI at age 5 years as independent variable.

**Table 3:** Health outcomes at age 7 years, predicted by BMI status at age 5 years.

		Underweight (n = 355)†	Normal weight (n = 1830)†	Overweight (n = 148)†	Obesity (n = 39)†
	n	OR (95% CI)	OR (95% CI)	OR (95% CI)	OR (95% CI)
One or more visits to GP (yes)^1^	2316	**2.64 (1.37; 5.00)** **	1.00	**3.41 (1.51; 7.69)** **	**13.39 (5.43; 33.03)** ***
Respiratory symptoms (yes) – wheezing^1^	2328	1.25 (0.79; 1.99)	1.00	1.57 (0.84; 2.94)	2.40 (0.91; 6.36)
Respiratory symptoms (yes) – dyspnea^1^	2322	1.06 (0.67; 1.67)	1.00	0.96 (0.47; 1.93)	1.74 (0.60; 5.04)
Hearing difficulties (yes)^1^	2301	1.14 (0.73; 1.78)	1.00	1.14 (0.60; 2.17)	1.18 (0.35; 3.94)
Seeing difficulties (yes)^1^	2295	1.32 (0.53; 3.26)	1.00	1.63 (0.48; 5.54)	4.48 (0.99; 20.34)
Abdominal pain (yes)^1^	2298	**1.44 (1.02; 2.03)** *	1.00	1.00 (0.57; 1.76)	1.00 (0.35; 2.89)
Headaches or migraine (yes)^1^	2296	1.25 (0.57; 2.74)	1.00	1.51 (0.52; 4.35)	1.40 (0.18; 10.71)
Allergies (yes)^1^	2301	**1.81 (1.32; 2.49)** ***	1.00	1.22 (0.71; 2.07)	1.07 (0.37; 3.08)
Eczema (yes)^1^	2292	0.65 (0.42; 1.00)	1.00	1.15 (0.68; 1.97)	0.72 (0.22; 2.38)
Lower score on general health^1^	2333	**1.47 (1.12; 1.94)** **	1.00	1.31 (0.87; 1.98)	**2.60 (1.32; 5.13)** **
Lower score on happiness^2^	2306	0.99 (0.77; 1.26)	1.00	1.21 (0.84; 1.75)	0.80 (0.41; 1.55)
Higher score on feeling insecure^2^	2330	1.12 (0.85; 1.47)	1.00	**6.37 (4.62; 8.78)** ***	**23.81 (13.05; 43.42)** ***
Higher score on adverse treatment^2^	2329	**1.39 (1.05; 1.84)** *	1.00	**5.70 (4.10; 7.92)** ***	**35.34 (19.16; 65.17)** ***
Parental concern^2^	2331	**1.84 (1.48; 2.30)** ***	1.00	**7.22 (5.31; 9.87)** ***	**26.10 (14.21; 47.94)** ***

^1^Odds ratio (OR) and 95% confidence interval (95% CI) from logistic regression analysis.

^2^Odds ratio (OR) and 95% confidence interval (95% CI) from ordinal regression analysis.

† Categories based on international age- and gender-specific BMI cut-off values. Note: all models are corrected for confounding by gender and ethnic background of the child and education level of the mother. Numbers printed **bold** represent significant OR and asterisks represent significance level;

* p<0.05,

** p<0.01,

*** p<0.001.

The OR for visiting the GP once or more was 2.64 (95% CI: 1.37 to 5.00) for underweight children. Compared to normal weight children, underweight children had an OR of 1.39 (95% CI: 1.05 to 1.84) for being treated adversely according to their parents. Their parents had an OR of 1.84 (95% CI: 1.48 to 2.30) for being concerned (Table 3).

For overweight children the OR for visiting the GP once or more was 3.41 (95% CI: 1.51 to 7.69). Compared to normal weight children, overweight children had an OR of 6.37 (95% CI: 4.62 to 8.78) for feeling insecure and 5.70 (95% CI: 4.10 to 7.92) for being treated adversely, as reported by their parents. Compared to normal weight children, the OR for parental concern was 7.22 (95% CI: 5.31 to 9.87) for overweight children (Table 3).

The OR for visiting the GP once or more was 13.39 (95% CI: 5.43 to 33.03) for obese children. Compared to the reports of parents of normal weight children, obese children had an OR of 35.34 (95% CI: 19.16 to 65.17) for being treated adversely. Compared to normal weight children, the OR was 26.10 (95% CI: 14.21 to 47.94) for parental concern about obese children (Table 3).

### Associations between Weight Trajectories and Health Outcomes at Age 7 Years

Table 4 presents the results for the regression model predicting health outcomes at age 7 years using the weight trajectories as independent variable.

**Table 4:** Health outcomes at age 7 years, predicted by weight trajectory between the age of 5 and 7 years.

		Low stable (n = 1732)	Increasing (n = 115)	High stable (n = 103)	Decreasing (n = 45)
	n	OR (95% CI)	OR (95% CI)	OR (95% CI)	OR (95% CI)
One or more visits to GP (yes)^1^	1951	1.00	0.88 (0.21; 3.74)	**3.87 (1.75; 8.54)** **	1.86 (0.42; 8.17)
Respiratory symptoms (yes) – wheezing^1^	1959	1.00	0.88 (0.38 2.07	1.93 (0.99; 3.76)	0.78 (0.19; 3.27)
Respiratory symptoms (yes) – dyspnea^1^	1953	1.00	0.37(0.11; 1.17)	0.99 (0.45; 2.20)	0.65 (0.16; 2.73)
Hearing difficulties (yes)^1^	1939	1.00	1.67 (0.89; 3.15)	1.52 (0.77; 3.04)	0.32 (0.04; 2.33)
Seeing difficulties (yes)^1^	1934	1.00	0.00 (0.00; –)	2.56 (0.74; 8.85)	1.86 (0.24; 14.21)
Abdominal pain (yes)^1^	1937	1.00	1.07 (0.58; 1.95)	1.06 (0.56; 1.99)	0.81 (0.29; 2.31)
Headaches or migraine (yes)^1^	1935	1.00	1.46 (0.44; 4.88)	2.23 (0.76; 6.54)	1.26 (0.17; 9.49)
Allergies (yes)^1^	1940	1.00	1.05 (0.58; 1.92)	1.24 (0.68; 2.28)	0.84 (0.30; 2.39)
Eczema (yes)^1^	1935	1.00	0.99 (0.52; 1.89)	0.98 (0.50; 1.94)	1.57 (0.68; 3.59)
Lower score on general health^1^	1964	1.00	1.09 (0.52; 2.29)	1.28 (0.79; 2.07)	1.14 (0.71; 1.84)
Lower score on happiness^2^	1940	1.00	1.10 (0.73; 1.67)	1.19 (0.76;1.85)	0.76 (0.41; 1.40)
Higher score on feeling insecure^2^	1961	1.00	**5.61 (3.88; 8.11)** ***	**16.35 (11.08; 24.36)** ***	**4.38 (2.48; 7.73)** ***
Higher score on adverse treatment^2^	1961	1.00	**4.72 (3.23;6.90)** ***	**15.94 (10.75; 23.64)** ***	**2.82 (1.53; 5.20)** ***
Parental concern^2^	1963	1.00	**5.55 (3.91; 7.89)** ***	**14.54 (9.91; 21.33)** ***	**3.61 (2.10; 6.18)** ***

^1^Odds ratio (OR) and 95% confidence interval (95% CI) from logistic regression analysis.

^2^Odds ratio (OR) and 95% confidence interval (95% CI) from ordinal regression analysis. Note: all models are corrected for confounding by gender and ethnic background of the child and education level of the mother. Numbers printed **bold** represent significant OR and asterisks represent significance level;

* p<0.05,

** p<0.01,

*** p<0.001.

Compared to children with a low stable weight, parents of children with an increasing weight trajectory reported significantly higher ORs for their child feeling insecure (OR: 5.61, 95% CI: 3.88 to 8.11) and being treated adversely (OR: 4.72, 95% CI: 3.23 to 6.90). Compared to parents of children with a low stable BMI, parents of children with an increasing weight trajectory reported a higher OR for concern (OR: 5.55, 95% CI: 3.91 to 7.89) (Table 4).

Compared to children with a low stable trajectory, children with a high-stable weight trajectory had a significantly higher OR for visiting the GP once or more (OR: 3.87, 95% CI: 1.75 to 8.54). Children with a high stable weight trajectory had higher ORs for feeling insecure (OR: 16.35, 95% CI: 11.08 to 24.36) and being treated adversely (OR: 15.94, 95% CI: 10.75 to 23.64), according to their parents. Compared to children with a low stable weight trajectory, parents of children with a high-stable weight trajectory showed a higher OR for parental concern (OR: 14.54, 95% CI: 9.91 to 21.33) (Table 4).

Compared to parents of children with a low stable weight trajectory, parents of children with a decreasing weight trajectory reported their child having a higher OR for feeling insecure (OR: 4.38, 95% CI: 2.48 to 7.73) and being treated adversely (OR: 2.82, 95% CI: 1.53 to 5.20). Compared to low stable weight children, parents of children with a decreasing weight reported more concern (OR: 3.61; 95% CI: 2.10 to 6.18) (Table 4).

## Discussion

This study first prospectively evaluated the association between the weight status of children aged 5 years and parent-reported health outcomes at age 7 years. The study shows that overweight and obese children visit the GP more often; there was no indication that overweight or obese children have more physical health conditions such as asthma symptoms or allergies. According to their parents, overweight and obese children experience more insecurity and are more often being treated adversely. Underweight children also appeared to experience more adverse treatment and lower general health. Parents of both underweight and overweight or obese children reported more concern about their child’s weight compared to parents of normal weight children.

Secondly this study evaluated the association of weight status trajectory, based on the weight status of the child at age 5 years and at age 7 years, with parent-reported health outcomes at child age 7 years. Overweight or obesity at 5 and 7 years of age was associated with more insecurity and adverse treatment compared to children with a normal weight at both ages.

### Associations between Overweight and Obesity, and Health Outcomes

According to their parents, overweight and obese children had a higher risk of feeling insecure or being treated adversely due to their weight. This concurs with other studies on children of a similar age (5–9 years) [18], [19], [20]. For example, one study that also evaluated the association between weight status and wellbeing in a longitudinal sample of general population children (but did not report on adverse treatment experienced by children), reported significantly higher odds for 4–5 year old overweight/obese children to have emotional and peer problems at age 8–9 years [19].

Parents of children with overweight and obesity reported more concern with regard to their child’s weight. Although our study did not indicate that overweight and obese children experience more health conditions (e.g. asthma symptoms, allergies), several health conditions are reported to be potentially associated with overweight and obesity (e.g. type 2 diabetes, sleep problems) [32]. Hypothetically, parents and children may have visited the GP for conditions unmeasured in the current study. Also, the smaller number of children with specific conditions may have created a lack of power to detect an effect of weight status in the current study. Nevertheless, other researchers have reported that parents are more aware and likely to identify the overweight of their 6-year-old child, compared to parents with younger children, and therefore concern with regard to the child’s weight may have increased among these parents [33].

### Associations between Underweight and Health Outcomes

Underweight children had slightly higher odds for being treated adversely compared to normal weight children. Two studies reported that adverse treatment was associated with both underweight and overweight [13] [15]. Although their results indicate that the odds for experiencing adverse treatment are much greater for overweight/obese children compared to the odds for underweight children, future studies will need to include underweight as a separate subgroup to further explore the associations with health outcomes.

Parents of underweight children reported slightly higher odds for higher levels of concern compared to parents of normal weight children. Also, a higher frequency of GP visits and lower scoring on the general health scale of the Child Health Questionnaire at age 7 years was observed. Hypothetically, underweight children may be more prone to seasonal diseases (such as influenza or a cold) which may partly explain the increased risk for visits to the GP and the overall lower scores on general health.

Although we observed some interesting associations between underweight in children and parent-reported health outcomes, these associations are to be interpreted with caution. We, for example, did not measure whether these children had specific diseases during preschool. Because children with relative underweight may develop a normal weight when they grow older [34], longitudinal data needs to provide more insight in weight patterns of these children. Health care practitioners may be attentive to health problems associated with childhood underweight so that appropriate advice can be given; however, more research is needed before reliable advice with regard to counseling for underweight children and their parents can be given.

### Associations between Weight Status Trajectories and Health Outcomes

Our study indicates that children with an increasing BMI between the age of 5 and 7 years have higher odds for being treated adversely and feeling insecure, as also reported by other studies [18], [19], [20]. Weight patterns have been associated with lower school functioning among elementary school-aged children [35]. The association between weight patterns and lower school functioning has been found to be mediated by internalizing factors (e.g. loneliness, low self-esteem) [35], [36]. This emphasizes the need to develop and evaluate appropriate interventions for overweight/obese children at young ages to prevent further decreases in school performance, social participation, health outcomes and quality of life. Also, the pathways and environmental characteristics through which health outcomes are affected by overweight or obesity need further clarification; qualitative studies are required to gain more insight into these mechanisms. Combining multiple resources, such as child, parent report and teacher reports, or performing observational studies, may help to elucidate the association between weight and health outcomes.

Based on the methods used in other studies we categorized children in weight status trajectories using the international cutoff values at age 5 and age 7 years, which may result in a relatively crude categorization [35], [36]. Children may decrease or increase within a weight category, but not reach the criterion to be categorized in another weight category. We explored whether gain in BMI was associated with higher risk for adverse psychosocial outcomes (data not shown). Children that gained BMI had a higher risk for being treated adversely and feeling insecure at age 7, as reported by their parents. Also, parents reported more concern with regard to their child’s weight. Considering the physical outcomes, children had a higher parent-reported OR for having one or more health conditions or visits to the GP when they gained BMI between age 5 and 7. This is in line with the results we observed using the trajectories approach for high stable and increasing weight status. Longitudinal studies having access to multiple BMI measures may be able to create individual pathways of BMI development using statistical models [37], [38], [39]. These longitudinal trajectories or developmental pathways may reveal more distinct patterns of for example, late or early onset BMI gain, and can be related to health outcomes [37], [38], [39].

### Methodological Considerations

Strengths of this study include the large sample size, the ability to create subgroups based on the international cut-off values for BMI, inclusion of a large group of underweight children and the availability of data at child age 5 years and child age 7 years.

Limitations include the missing data at child age 7 years and parents self-report of the children’s health outcomes. Also, mothers of children with complete outcome measures differ from mothers with missing outcome data; however, this does not necessarily influence the associations under study. With regard to the items used to measure psychosocial health outcomes of the child, these have not been examined with regard to validity and reliability. Additional analyses (data not shown) were performed to gain insight in the validity of the items used. These analyses showed that normal weight mothers reported a higher OR for their overweight or obese child to be treated adversely and feel insecure, normal weight mothers also reported more concern for their overweight or obese child compared to normal weight children (data not shown). Nevertheless, we recommend future research to evaluate the validity and reliability of the items measuring psychosocial health outcomes.

The use of parent self-report may have led to over- or underestimation of the children’s health outcomes and needs to be taken into account when interpreting the findings. Measures of depression and self-esteem of the child were not included in the questionnaire because of the already reasonably high respondent burden. Although child report may have provided more accurate estimates of consequences on health outcomes, measuring concepts such as self-esteem and depression is known to be challenging among young children [40]. Also, at younger age self-concept indicators, such as teasing and insecurity, may be more informative compared to self-esteem questionnaires due to the developmental stage of the children [40].

### Conclusions

In conclusion, parents reported their overweight, obese and underweight children to be more often treated adversely or feel insecure due to their weight. Parents of overweight, obese and underweight children expressed more concern about health outcomes associated with their child’s overweight or underweight. These concerns seem to be reflected in the more frequent parent-reported visits to the GP of children with overweight, obesity and underweight.

Future studies need to follow-up on the associations between weight status and health outcomes when children develop and reach adolescence and adulthood. Also, underlying mechanisms and pathways associated with weight status and health outcomes need to be assessed in, preferably, longitudinal research.

In the meantime, we recommend that healthcare providers be alert to early signs of adverse treatment and insecure feelings in both overweight and underweight children. Appropriate counseling for teasing and insecure feelings should be offered in addition to, or as part of, interventions aiming at a positive change in weight status.

## Supporting Information

Figure S1 **Flow chart of the data selection process.**
(TIF)

Table S1 Baseline percentages of children visiting the GP and children experiencing certain health conditions; parent report.
(DOC)

Table S2 Results of stratified regression analyses predicting health outcomes at age 7 years with BMI-status at age 5 years as predictor.
(DOC)
