# Supplementary material for: Overweight, Obesity and Underweight Is Associated with Adverse Psychosocial and Physical Health Outcomes among 7-Year-Old Children: The ‘Be Active, Eat Right’ Study
Source: PLoS One. 2013 Jun 25;8(6):e67383. doi: 10.1371/journal.pone.0067383 (PMC3692418; doi:10.1371/journal.pone.0067383)
Supplement: Table S1 — Baseline percentages of children visiting the GP and children experiencing certain health conditions; parent report. (DOC) [file pone.0067383.s002.doc]

Table S1 Baseline percentages of children visiting the GP and children experiencing certain health conditions; parent report

|  | n | Total (n=3912) | Underweight (n=595)† | Normal weight (n=2956)† | Overweight (n=288)† | Obesity (73)† | p-value |
| --- | --- | --- | --- | --- | --- | --- | --- |
| Visits to GP or specialist (% yes) | 2651 | 59.4 | 59.8 | 58.5 | 64.7 | 79.1 | **0.020**** |
| Respiratory symptoms (% yes) – wheezing | 2927 | 9.1 | 7.9 | 9.1 | 9.3 | 18.0 | 0.131** |
| Respiratory symptoms (% yes) – dyspnea | 2904 | 9.3 | 7.7 | 9.6 | 7.5 | 16.3 | 0.154** |
| Hearing difficulties (% yes) | 2873 | 10.4 | 10.9 | 10.1 | 15.3 | 2.2 | **0.033*** |
| Seeing difficulties (% yes) | 2877 | 1.3 | 1.6 | 1.3 | 1.0 | 0 | 0.800* |
| Abdominal pain (% yes) | 2871 | 9.6 | 10.5 | 9.6 | 8.9 | 4.4 | 0.592* |
| Headaches or migraine (% yes) | 2874 | 1.1 | 0.9 | 1.2 | 1.5 | 0 | 0.804* |
| Allergies (% yes) | 2886 | 9.4 | 11.6 | 9.0 | 7.0 | 16.7 | 0.062* |
| Eczema (% yes) | 2883 | 12.2 | 9.8 | 12.8 | 10.3 | 17.4 | 0.181* |

† Categories based on international age- and gender-specific BMI cut-off values

Note: Asterisks represent type of analysis used to compare weight categories: * Kruskal-Wallis, ** Chi-square, *** ANOVA

Numbers printed **bold** represent significant p-value
