# Supplementary material for: Overweight, Obesity and Underweight Is Associated with Adverse Psychosocial and Physical Health Outcomes among 7-Year-Old Children: The ‘Be Active, Eat Right’ Study
Source: PLoS One. 2013 Jun 25;8(6):e67383. doi: 10.1371/journal.pone.0067383 (PMC3692418; doi:10.1371/journal.pone.0067383)
Supplement: Table S2 — Results of stratified regression analyses predicting health outcomes at age 7 years with BMI-status at age 5 years as predictor. (DOC) [file pone.0067383.s003.doc]

**Table S2 Results of stratified regression analyses predicting health outcomes at age 7 years with BMI-status at age 5 years as predictor**

|  |  |  | Underweight† | Normal weight† | Overweight† | Obesity† |
| --- | --- | --- | --- | --- | --- | --- |
|  |  | n | OR (95% CI) | OR (95% CI) | OR (95% CI) | OR (95% CI) |
| One or more visits to GP (yes)¹ | Boy | 1173 | **4.03 (1.51;10.74)**** | 1.00 | **7.57 (2.29; 24.99)**** | **46.35 (13.40; 160.37)***** |
|  | Girl | 1180 | 2.08 (0.90; 4.83) | 1.00 | 2.09 (0.70; 6.29) | **7.19 (1.96; 26.37)**** |
|  | Dutch | 2088 | **2.45 (1.19; 5.05)*** | 1.00 | **3.34 (1.34; 8.32)*** | **9.10 (2.55; 32.47)**** |
|  | Non-Dutch | 231 | **4.03 (1.02; 15.82)*** | 1.00 | 4.03 (1.02; 15.82) | **26.83 (6.09; 118.29)***** |
|  | Low educated | 453 | 0.74 (0.09; 6.09) | 1.00 | **4.631 (1.14; 18.81)*** | 4.79 (0.54; 42.66) |
|  | High educated | 1895 | **3.41 (1.72; 6.74)***** | 1.00 | **3.14 (1.17; 8.47)*** | **24.32 (9.28; 63.72)***** |
| Respiratory symptoms- wheezing (yes)¹ | Boy | 1180 | 1.47 (0.80; 2.71) | 1.00 | 1.31 (0.46; 3.77) | **5.04 (1.59; 15.99)**** |
|  | Girl | 1186 | 1.02 (0.51; 2.06) | 1.00 | 1.70 (0.78; 3.72) | 0.85 (0.11; 6.46) |
|  | Dutch | 2097 | 1.15 (0.69; 1.91) | 1.00 | 1.48 (0.75; 2.91) | **3.22 (1.09; 9.55)*** |
|  | Non-Dutch | 235 | 1.98 (0.65; 6.01) | 1.00 | 1.87 (0.38; 9.20) | 1.19 (0.14; 10.01) |
|  | Low educated¶ | 460 | 0.52 (0.15; 1.77) | 1.00 | 0.33 (0.04; 2.52) | 0.00 (0.00; …)¤ |
|  | High educated¶ | 1900 | 1.52 (0.92; 2.51) | 1.00 | **2.11 (1.09; 4.11)*** | **4.58 (1.68; 12.48)**** |
| Respiratory symptoms- dyspnea (yes)¹ | Boy | 1176 | 1.07 (0.56; 2.03) | 1.00 | 1.11 (0.39; 3.17) | 2.97 (0.83; 10.62) |
|  | Girl | 1183 | 0.99 (0.52; 1.88) | 1.00 | 0.82 (0.32; 2.10) | 0.68 (0.09; 5.13) |
|  | Dutch | 2094 | 0.97 (0.59; 1.58) | 1.00 | 1.05 (0.52; 2.12) | 2.56 (0.87; 7.55) |
|  | Non-Dutch | 231 | 1.82 (0.54; 6.16) | 1.00 | 0.00 (0.00; …)¤ | 0.00 (0.00; …)¤ |
|  | Low educated | 456 | 0.33 (0.08; 1.42) | 1.00 | 0.65 (0.15; 2.86) | 0.00 (0.00; …)¤ |
|  | High educated | 1898 | 1.25 (0.77; 2.03) | 1.00 | 1.01 (0.46; 2.24) | 2.73 (0.92; 8.10) |
| One or more conditions (yes)§¹ | Boy | 840 | 1.33 (0.91; 1.93) | 1.00 | 1.04 (0.54; 2.00) | 1.61 (0.55; 4.70) |
|  | Girl | 858 | 0.93 (0.65; 1.33) | 1.00 | 0.94 (0.57; 1.55) | 1.08 (0.41; 2.81) |
|  | Dutch | 1787 | 1.12 (0.85; 1.47) | 1.00 | 1.08 (0.71; 1.64) | 1.75 (0.74; 4.15) |
|  | Non-Dutch | 195 | 0.89 (0.39;2.94) | 1.00 | 0.51 (0.13; 1.93) | 0.73 (0.18; 2.94) |
|  | Low educated | 387 | 1.05 (0.58; 1.91) | 1.00 | 0.67 (0.27; 1.62) | 2.00 (0.57; 7.07) |
|  | High educated | 1620 | 1.12 (0.84; 1.49) | 1.00 | 1.13 (0.73; 1.77) | 1.08 (0.45; 2.59) |

***Table S1*** continued

|  |  |  | Underweight† | Normal weight† | Overweight† | Obesity† |
| --- | --- | --- | --- | --- | --- | --- |
|  |  | n | OR (95% CI) | OR (95% CI) | OR (95% CI) | OR (95% CI) |
| Lower scores for general health² | Boy | 1184 | **1.75 (1.20; 2.55)**** | 1.00 | 1.46 (0.77; 2.78) | 2.57 (0.94; 7.04) |
|  | Girl | 1186 | 1.15 (0.77; 1.72) | 1.00 | 1.22 (0.72; 2.08) | **3.29 (1.38; 7.84)**** |
|  | Dutch | 2100 | **1.45 (1.09; 1.95)*** | 1.00 | 1.10 (0.70; 1.75) | 1.79 (0.75; 4.31) |
|  | Non-Dutch | 236 | 1.46 (0.65; 3.31) | 1.00 | **3.27 (1.20; 8.94)*** | **5.73 (1.71; 19.19)**** |
|  | Low educated | 464 | 0.97 (0.49; 1.91) | 1.00 | 0.28 (0.56; 2.96) | **5.26 (1.71; 16.19)**** |
|  | High educated | 1901 | **1.56 (1.15; 2.11)**** | 1.00 | 1.32 (0.82; 2.11) | 2.15 (0.92; 5.00) |
| Lower scores for happiness² | Boy¤ | 1166 | **1.51 (1.04;2.18)*** | 1.00 | 0.71 (0.41; 1.23) | 0.69 (0.26; 1.83) |
|  | Girl¤ | 1177 | **0.72 (0.52;1.00)*** | 1.00 | 1.62 (0.99; 2.66) | 0.66 (0.28; 1.55) |
|  | Dutch | 2074 | 0.92 (0.71; 1.19) | 1.00 | 0.66 (0.30; 1.46) | 1.26 (0.85; 1.89) |
|  | Non-Dutch | 235 | 2.00 (0.93; 4.29) | 1.00 | 0.64 (0.32; 1.67) | 1.06 (0.32; 3.46) |
|  | Low educated¶ | 461 | 1.36 (0.80; 2.33) | 1.00 | 1.14 (0.57; 2.28) | 0.45 (0.15; 1.38) |
|  | High educated¶ | 1877 | 0.93 (0.71; 1.22) | 1.00 | 1.20 (0.78; 1.85) | 0.92 (0.40; 2.10) |
| Higher scores for feeling insecure² | Boy | 1183 | **1.51 (1.01; 2.25)*** | 1.00 | **5.67 (3.35; 9.61)***** | **35.30 (14.45; 86.31)***** |
|  | Girl | 1186 | 0.88 (0.61; 1.28) | 1.00 | **7.00 (4.67; 10.49)***** | **19.63 (8.97; 42.99)***** |
|  | Dutch¶ | 2098 | 1.26 (0.95; 1.68) | 1.00 | **8.22 (5.85; 11.58)***** | **19.24 (9.47; 39.06)***** |
|  | Non-Dutch¶ | 237 | 0.51 (0.21; 1.24) | 1.00 | 2.21 (0.88; 5.58) | **29.93 (9.09; 98.59)***** |
|  | Low educated | 462 | 0.68 (0.35; 1.30) | 1.00 | **6.40 (3.36; 12.22)***** | **12.48 (4.51; 34.57)***** |
|  | High educated | 1901 | 1.28 (0.95; 1.72) | 1.00 | **6.96 (4.83; 10.03)***** | **34.19 (16.54; 70.74)***** |
| Higher scores for adverse treatment ² | Boy¤ | 1183 | **1.75 (1.18; 2.58)***** | 1.00 | **3.58 (2.05; 6.25)***** | **44.12 (17.85; 108.96)***** |
|  | Girl¤ | 1184 | 1.11 (0.75; 1.64) | 1.00 | **8.00 (5.26; 12.13)***** | **34.19 (15.35; 76.25)***** |
|  | Dutch | 2097 | **1.52 (1.13; 2.04)**** | 1.00 | **6.86 (4.83; 9.74)***** | **37.41 (18.12; 84.44)***** |
|  | Non-Dutch | 236 | 0.79 (0.35; 1.81) | 1.00 | **3.03 (1.20; 7.61)*** | **24.34 (7.68; 77.01)***** |
|  | Low educated | 462 | 0.96 (0.52; 1.77) | 1.00 | **3.81 (1.97; 7.35)***** | **17.50 (6.23; 49.21)***** |
|  | High educated | 1899 | **1.54 (1.13; 2.10)**** | 1.00 | **7.09 (4.86; 10.32)***** | **48.91 (23.45; 102.00)***** |

***Table S1*** continued

|  |  |  | Underweight† | Normal weight† | Overweight† | Obesity† |
| --- | --- | --- | --- | --- | --- | --- |
|  |  | n | OR (95% CI) | OR (95% CI) | OR (95% CI) | OR (95% CI) |
| Parental concern² | Boy | 1183 | **2.04 (1.48; 2.81)***** | 1.00 | **7.51 (4.56; 12.37)***** | **43.42 (17.39; 108.31)***** |
|  | Girl | 1185 | **1.64 (1.21; 2.23)***** | 1.00 | **7.32 (4.91; 10.89)***** | **19.39 (8.82; 42.69)***** |
|  | Dutch | 2098 | **1.96 (1.55; 2.48)***** | 1.00 | **8.42 (6.04; 11.74)***** | **28.53 (13.85; 58.79)***** |
|  | Non-Dutch | 236 | 1.21 (0.62; 2.37) | 1.00 | **3.76 (4.23; 9.09)**** | **16.76 (5.42; 51.83)***** |
|  | Low educated | 462 | 1.33 (0.80; 2.21) | 1.00 | **5.83 (3.10; 10.98)***** | **24.31 (8.50; 69.48***** |
|  | High educated | 1900 | **1.97 (1.54; 2.51)***** | 1.00 | **8.25 (5.78; 11.78)***** | **28.30 (13.68; 58.62)***** |

¹ Odds ratio (OR) and 95% confidence interval (95% CI) from logistic regression analysis

² Odds ratio (OR) and 95% confidence interval (95% CI) from ordinal regression analysis

§ Chronic conditions was dichotomized into none versus one or more conditions for the stratified analyses

† Categories based on international age- and gender-specific BMI cut-off values

¶ Significant interaction term (weight category times potential moderating variable) in regression model corrected for potential confounding variables p<0.10

¤ OR could not be computed due to low cell counts

Note: No correction for potential confounding variables in stratified analyses. Education level of the mother was dichotomized due to low cell counts in the stratified analyses. Numbers printed **bold** represent significant ORs and asterisks represent significance level; * p< 0.05, **p< 0.01, ***p< 0.001
